# The Influence of Different Bleaching Protocols on Dentinal Enzymatic Activity: An In Vitro Study

**DOI:** 10.3390/molecules27051684

**Published:** 2022-03-04

**Authors:** Eric Mayer-Santos, Tatjana Maravic, Allegra Comba, Patricia Moreira Freitas, Giovanna Bueno Marinho, Claudia Mazzitelli, Edoardo Mancuso, Nicola Scotti, Federica Florenzano, Lorenzo Breschi, Annalisa Mazzoni

**Affiliations:** 1 Department of Restorative Dentistry, School of Dentistry, University of São Paulo, São Paulo 05508-000, Brazil; eric.mayer.santos@alumni.usp.br (E.M.-S.); pfreitas@usp.br (P.M.F.); giovannamarinho@usp.br (G.B.M.); 2 Department of Biomedical and Neuromotor Sciences, University of Bologna, 40125 Bologna, Italy; tatjana.maravic@unibo.it (T.M.); claudia.mazzitelli@unibo.it (C.M.); edoardo.mancuso2@unibo.it (E.M.); federica.florenzano3@unibo.it (F.F.); lorenzo.breschi@unibo.it (L.B.); 3 Department of Surgical Sciences, University of Turin, 10126 Turin, Italy; allegra.comba@unito.it (A.C.); nicola.scotti@unito.it (N.S.)

**Keywords:** tooth whitening, bleaching, zymography, matrix metalloproteinases

## Abstract

This study aimed to investigate matrix metalloproteinase (MMP) activity in human dentin using in-situ and gelatin zymography, after at-home and in-office bleaching, related to their clinical exposure times. Dentin specimens (*n* = 5) were treated with 35% hydrogen peroxide (50 min per session/4 sessions), 10% carbamide peroxide (180 min/21 sessions), or no treatment. All were subjected to in-situ zymography. Dentin slices were, subsequently, obtained, covered with fluorescein-conjugated gelatin, and examined with confocal laser-scanning microscopy. The fluorescence intensity was quantified and statistically analyzed using one-way ANOVA and Bonferroni tests (α = 0.05). Furthermore, gelatin zymography was performed on protein extracts obtained from dentin powder (*N* = 8 teeth), treated with hydrogen peroxide or carbamide peroxide, with different exposure times (10/50 min for hydrogen peroxide; 252/1260 min for carbamide peroxide). The results of the in-situ zymography showed no statistical differences between the bleached specimens and the control group, with a medium level of gelatinolytic activity expressed in the dentin tubules. The results of gelatin zymography showed an increased expression of pro-MMP-9 in carbamide peroxide groups. The expression of pro-MMP-2 decreased in all the experimental groups. The bleaching treatments performed on the enamel of sound teeth do not influence dentinal enzymatic activity. However, when unprotected dentin tissue is bleached, matrix metalloproteinases are more expressed, particularly when carbamide peroxide is used, proportional to the exposure time.

## 1. Introduction

Esthetics has gained growing recognition in dentistry, reflecting an increasing demand of patients who consider the smile a factor of great relevance [1,2]. One of the most important aspects regarding tooth appearance is its color, and dental bleaching is a conservative technique that can remove discolorations from the tooth tissues. In this context, professionals are acutely aware of the importance of tooth bleaching in the clinical practice [3,4,5,6].

Currently, two professionally supervised techniques are commonly reported: the in-office and the at-home bleaching. The most commonly used active ingredients are hydrogen peroxide (HP) and carbamide peroxide (CP), applied in different concentrations, depending on the application method [4,6]. Bleaching occurs as a result of the breakdown of pigmented molecules located in the enamel and dentin, caused by reactive oxygen species (ROS) released from bleaching agents, such as hydroxyl radicals (OH^−^) and singlet oxygen (^1^O_2_) [7]. These chemicals rapidly diffuse through the enamel and dentin and, according to some authors, could cause changes in the elastic modulus, microhardness, and morphology of hard dental tissues [4,5]. Clinically, patients often report dental sensitivity. Moreover, some studies suggest induced chemical degradation within the collagen fibrils, which can cause oxidative agents to denature proteins in dentin [5,8].

The dentin matrix is a complex network of fibrillar and globular structures that form the organic scaffold of dentin. Matrix metalloproteinases (MMPs) and cysteine cathepsins (CC) are Ca^2+^/Zn^2+^-dependent endopeptidases of the extracellular matrix, involved in numerous physiological and pathological processes. It is assumed that these enzymes have active roles in tooth formation, and, after the mineralization of dentin, they remain “fossilized” and, hence, inactive. The MMPs secreted by the odontoblasts, however, have a lifelong role in the response of tooth tissues to inflammation or other exogenous factors. Furthermore, the inactive dentinal MMPs can be reactivated by different processes, such as caries and dental restorative procedures. Actually, various dental materials (etchants, adhesive resins, primers, etc.) were proven to cause an increase in the activity of the MMPs [9,10,11,12,13,14]. In this case, they have been shown to have an active role in the degradation of the extracellular matrix, influencing the longevity of resin–dentin restorations and secondary caries onsets [15,16,17,18,19]. Hence, it would be of high importance to investigate, in depth, how the bleaching agents influence the endogenous dentinal enzymatic activity. Usually, bleaching agent is applied on enamel. However, there are certain clinical situations when bleaching is performed on a section of exposed dentin, and both these clinical situations should be considered. It has been reported that bleaching treatments may increase the MMP-mediated collagen degradation in dentin [8,20]. However, to the best of our knowledge, there are no studies investigating the activity of MMPs by means of gelatin zymography and in-situ zymography, after at-home and in-office bleaching protocols with their clinical exposure times. Clinically, it is important to evaluate the unexplored possible side effects of the treatment.

Accordingly, the objective of this study was to evaluate, qualitatively and quantitatively, the influence of both at-home and in-office bleaching treatments on dentinal enzymatic activity using in-situ zymography and gelatin zymography. The null hypotheses in this study were: (1) in-office and at-home bleaching through the enamel does not influence dentinal enzymatic activities; and (2) the treatment of dentin powder with bleaching agents at different exposure times does not influence the expression of MMP-2 and -9.

## 2. Results

### 2.1. In-Situ Zymography

**Figure 1:**
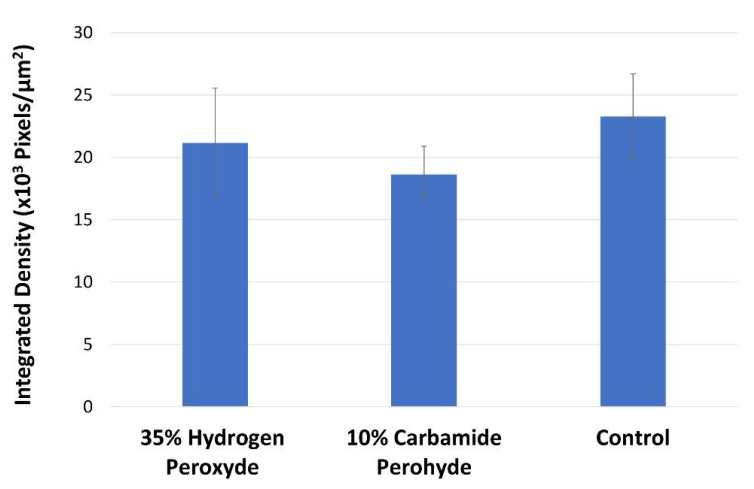
Graph of the integrated density of the fluorescent signal evaluated in-situ in different treatment groups (mean ± SE).

**Figure 2:**
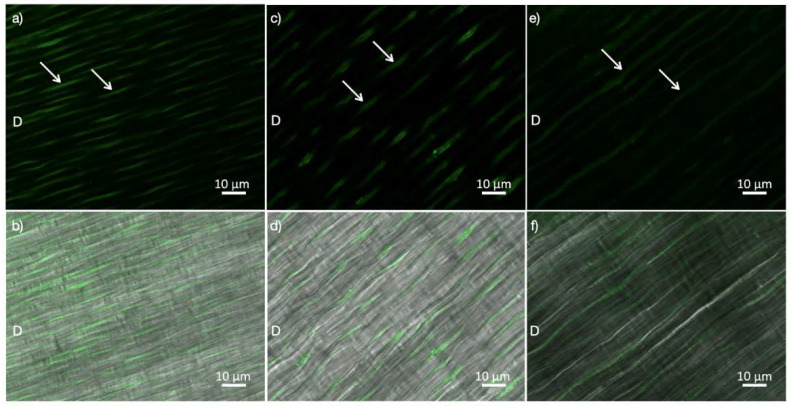
Dentin slices derived from all groups that were incubated with quenched fluorescein-labeled gelatin (D). In-situ zymographic assay showed similar enzymatic activity between the investigated groups. (**a**) Image acquired in the green channel, showing fluorescence (identifying medium level of endogenous enzymatic activity) in dentinal tubules induced with 35% HP treatment. (**b**) Image of 35% HP group, obtained by merging the differential interference contrast (DIC) image and the image acquired in the green channel. (**c**) Image acquired in the green channel, showing fluorescence (identifying medium level of endogenous enzymatic activity) in dentinal tubules induced with 10% CP treatment. (**d**) Image of 10% CP group, obtained by merging the DIC image and the image acquired in the green channel. (**e**) Image acquired in the green channel, showing fluorescence (identifying medium level of endogenous enzymatic activity) in dentinal tubules with no treatment (control group). (**f**) Image of the control group, obtained by merging the DIC image and the image acquired in the green channel. Arrows show enzymatic activity. HP: hydrogen peroxide, CP: carbamide peroxide.

The enzymatic activity (mean ± SD of the integrated density of the fluorescence signal) of the investigated groups is described in Figure 1. No statistically significant differences were observed (*p* > 0.05; ANOVA). The intensity of fluorescence, corresponding to the level of enzymatic activity within the dentin structure, can be observed in Figure 2. A medium level of gelatinolytic activity was noted within the dentinal tubules, while a low level of activity was observed in the intertubular dentin in all the groups.

### 2.2. Gelatin Zymography

**Figure 3:**
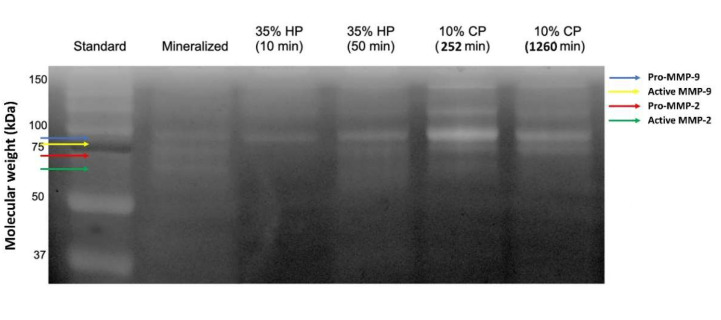
Zymography of protein extracts obtained from dentin powder. Lane 1: standards (Std) in kDa; Lane 2: mineralized untreated dentine powder; Lane 3: dentin powder treated with hydrogen peroxide (HP) 35% for 10 min; Lane 4: dentin powder treated with HP 35% for 50 min; Lane 5: dentin powder treated with carbamide peroxide (CP) at 10% for 252 min; Lane 6: dentin powder treated with CP 10% for 1260 min. Different colored arrows mark the molecular weight of different MMPs.

**Figure 4:**
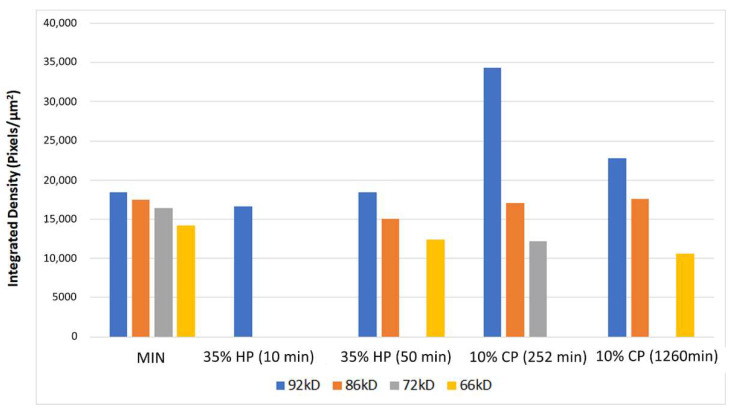
Graph illustrating the densitometric evaluation of bands obtained from the gelatin zymographic analysis of proteins extracted from dentin powder; 92 kDa molecular weight corresponds to pro-MMP-9; 86 kDa molecular weight corresponds to the active form of MMP-9; 72 kDa molecular weight corresponds to pro-MMP-2; 66 kDa molecular weight corresponds to the active form of MMP-2. HP: hydrogen peroxide, CP: carbamide peroxide.

The zymograms of gelatinolytic activity are shown in Figure 3. The band of mineralized sound dentin showed the pro-forms and active-forms of MMP-2 and -9 (72/66 and 92/86 kDa, respectively). The expression of MMP-9 remained similar to the control group after the bleaching treatment with 35% HP in both exposure times. On the other hand, the expression of the pro-form of MMP-9 increased markedly in the 10% CP groups, especially after the protocol of 252 min, which presented a more intense band. The expression of the pro-and active-forms of MMP-2 decreased in all the tested groups, with a complete inhibition in the 35% HP groups and a lower expression in the 10% CP groups, especially in the protocol of the 1260 min application (Figure 4).

## 3. Discussion

The present study evaluated the effects of different bleaching protocols on dentinal enzymatic activity using, for the first time, both in-situ zymography and gelatin zymography. Since the results demonstrated that there were no differences in the dentinal enzymatic activity between the control and experimental groups when the bleaching was applied through the enamel, the first null hypothesis was accepted. On the other hand, the gelatin zymography of the dentin powder showed differences in the expression of the MMPs between the groups, warranting the rejection of the second hypothesis.

The in-situ zymography was performed, aiming to investigate the more clinical approach, applying the bleaching treatment onto the enamel and evaluating the effects on dentin tissue. It is known that bleaching gels diffuse rapidly through the enamel and dentin, and could cause changes in the structure of dentin and pulp tissues, possibly leading to adverse effects [5,6]. In this context, it is important to know whether the enamel has a protective effect on the underlying tooth structure after the application of bleaching chemicals. On the other hand, gelatin zymography was used to obtain a quantification of the expression of specific pro- and active-forms of MMPs and to evaluate the influence of bleaching applied directly to the dentin structure since, clinically, bleaching agents are applied directly to dentin in endodontically treated teeth, or in case of non-carious cervical lesions.

MMPs are Ca/Zn-dependent endopeptidases implicated in physiological tissue remodeling, extracellular matrix degradation, tumor growth, and tumor invasion. Moreover, their activity has been attributed to several developmental events involving dental tissues, pathological periodontal processes, dental pulp inflammation, and caries [21,22,23,24,25]. As a collagen-based tissue, dentin contains several MMPs, such as gelatinases MMP-2 and MMP-9, collagenase MMP-8, enamelysin MMP-20, matrilysin MMP-7, and stromelysin MMP-3, which play active roles during teeth development [26,27,28,29,30]. However, MMPs are covered with apatite nanocrystals during dentin matrix mineralization, leaving them inactive. These proteases remain structurally stable as long as dentin is mineralized, but they can be reactivated after various dental procedures, due to caries, or local radiation therapy [31,32,33,34,35,36,37]. The activation of the MMPs occurs through a conformational change in the active site of the enzyme that causes the release of the cysteine residue from the “cysteine switch” and allows the activity of the catalytic site and the cleavage of the substrate [23,38]. It has been demonstrated that the interplay between ROS and MMPs can be rather complex. Namely, one of the modes of MMP pro-form activation is the modification of the free cysteine by ROS. However, by extended exposure, ROS can also inactivate the MMPs [38]. In this context, it is important to evaluate the effects of chemical bleaching agents on the activation of specific MMPs, such as MMP-2 and MMP-9, which can influence the future progression of carious lesions and the adhesion of resinous materials to the bleached substrate.

Based on in-situ zymography results, there were no differences in the dentinal enzymatic activity between the control and experimental groups, regardless of the concentration and pH of the different bleaching agents used. This could be due to the protective effect of the enamel, which was sufficient to avoid the enzymatic activation in the underlying dentin. Recently, Karaarslan et al. [7] performed an in situ study and evaluated, spectrofluorometrically, cathepsin B activation in the dentin powder and pulpal tissues, as well as the collagenolytic/gelatinolytic MMP activities. The authors found no alterations of cathepsin B and MMP proteolytic activities after different bleaching protocols applied directly on the enamel. Hence, their results are in accordance with the present study.

Current bleaching techniques differ in terms of the type, concentration, exposure time, product presentation, and mode of application [2,4,5,6,39]. The active bleaching component is the HP in both in-office and at-home bleaching, which can be applied directly or produced in a chemical reaction from CP. In the decomposition of a 10% CP concentration, 3.5% of HP is generated. In this context, CP is commonly indicated for at-home bleaching due to its high chemical stability, which allows its use for an extended period of time [6,40]. On the other hand, HP is frequently used for in-office bleaching, owing to its higher concentration and faster results. However, the concentration is directly related to the side effects, and the higher concentration gel increases the chances of undesirable effects, such as dental sensitivity, as well as changes in the elastic modulus, microhardness, and morphology of hard dental tissues [4,5,40]. For that reason, the present study was the first to use gelatin zymography to evaluate the bleaching effects on MMP-2 and -9 using different exposure times for both CP and HP. The exposure times of the dentin powder to the bleaching agents corresponded to one week of each treatment (50 min of HP and 1260 min of CP) and to a proportionally shorter (1/5) exposure time for both treatments (10 min of HP and 252 min of CP), providing a more comprehensive answer regarding the behavior of these chemicals on MMP activity, also in terms of the influence of application time.

The expression of gelatinases MMP-2 and -9 tested in the present study differed with regard to bleaching treatment and exposure time applied. MMP-9, particularly of the pro-form, showed an increased gelatinolytic activity after the application of 10% CP, especially in the protocol with the higher exposure time. Toledano et al. [8] reported an increase in MMP-mediated dentin collagen degradation, but not related specifically to MMP-9 or MMP-2. The increase in MMP-9 expressions observed in the current study supports their results, since its activation is directly linked to the degradation of the dentinal extracellular matrix [8,41,42]. ROS also promoted higher MMP-9 levels in cancer cells [43]. On the other hand, the expression of the MMP-2 pro-form decreased in all the bleached groups, particularly in the 35% HP ones. Viappiani et al. [44] demonstrated that the regulation of MMP-2 by the ROS is concentration dependent, with lower concentrations of ROS inducing the activation of the MMPs, and the higher concentrations causing MMP-2 inhibition. This could corroborate the results of the present study. Ten percent CP promoted a higher expression of MMP-9 in comparison to 35% HP, implying that this activation is not related to the pH, since the HP chemicals present a more acidic pH in comparison to CP. Regarding the behavior of CP, the lower exposure time protocol promoted a higher activation of MMP-9. These findings show, for the first time, how different dentinal MMPs react to the different exposure times of at-home bleaching chemicals. We could hypothesize that the increase in the enzymatic activity in the CP 252 min group could be related to the exposure time of dentin to the ROS released from the bleaching agents. The decrease in the gelatinolytic activity in the CP 1260 min group (although still high) could mean that, after a certain time of exposure, the dentinal enzymes can be partially degraded or their catalytic sites can be altered by the ROS and, hence, their expression is decreased [45,46]. Clinically, these results additionally support the literature that indicates in-office bleaching for patients presenting teeth with exposed dentin [6,47,48], since the at-home bleaching procedure showed a greater influence on the dentinal enzymatic activity, and was performed without gingival and cervical recession protection.

The limitation of the present study could be that the human teeth employed in the study were mainly healthy third molars, often extracted operatively, without ever being in occlusal function. Dentin is a very variable tissue that changes during the course of life due to the exposure to the oral conditions, mastication, and microbial attacks [49,50]. In the case studied in the present research, one of the rationales of the study was to investigate the influence of bleaching on the endogenous enzymatic dentinal activity in cases when dentin is exposed in the oral cavity. However, a very important point to consider is that, in this case, the dentin tissue would likely be sclerotic and, therefore, might have different interactions with the bleaching agent in terms of MMP expression and activity. A study by this research group is underway to disentangle this matter.

The results of the present study reaffirmed dental bleaching as a safe procedure, also in terms of endogenous dentinal enzymatic activity, if performed according to the recommended protocols, highlighting the importance of protecting exposed dentin tissues before applying bleaching chemicals. However, all the treatments in the present study were performed on healthy unrestored teeth, and further investigations need to be done in order to evaluate the effects of these procedures on teeth restored with direct composite resin fillings.

## 4. Materials and Methods

### 4.1. In-Situ Zymography

Crowns of non-carious freshly extracted human teeth (*n* = 5, sample size calculated using G*Power 3.1.9.7 for Windows with a calculated effect size of f = 1.0269636, α error probability = 0.05, power (1-β error probability) = 0.85) (Christian-Albrechts-University, Kiel, Germany), either stored in water at 4 °C and used within one week, or washed under running water and frozen immediately (−20 °C) and defrosted right before the protocol, were used for the present test. The tooth crowns were separated from the root and cut into four parts (two cuts perpendicular to each other on the occlusal surface) using a slow-speed diamond saw (Micromet, Remet, Bologna, Italy) with water cooling. Three parts from each tooth were used in the study. A blinded researcher (not aware of the testing groups) randomly assigned the tooth slices to 3 different groups (Table 1). The dentin exposed after cutting was protected with nail varnish, and the bleaching was performed on the enamel surface according to the different protocols (Table 1). The characteristics of 35% HP gel and 10% CP gel are described in Table 2.

After the bleaching protocols, the tooth quarters were cut into 1-mm thick specimens, glued to glass slides, and polished up to a thickness of approximately 50 µm. In-situ zymography was performed on the specimens immediately after the treatment, in accordance with Mazzoni et al. [10,15]. To produce the substrate, 1.0 mg/mL of a stock solution containing self-quenched fluorescein-conjugated gelatin (E-12055, Molecular Probes, Eugene, OR, USA) was prepared by adding 1.0 mL of deionized water to the vial containing the lyophilized gelatin. The gelatin stock solution was diluted 10 times using a dilution buffer (NaCl 150 mM, CaCl_2_ 5 mM, and Tris-HCl 50 mM, with a pH of 8.0). Fifty microliters of the fluorescent gelatin mixture were placed on the top of each polished tooth specimen and was protected with a cover slip. The glass slide assemblies were light-protected and were incubated in a humidified chamber at 37 °C overnight.

The detection of the endogenous gelatinolytic enzyme activity within the dentin structure was based on the hydrolysis of the quenched fluorescein-conjugated gelatin substrate. The process was evaluated by examining the glass slides with a multi-photon confocal laser scanning microscope (Leica SP8, Leica Microsystems GmbH, Wetzlar, Germany), using an excitation wavelength of 495 nm and an emission wavelength of 515 nm.

The optical sections (z-stacks of images comprising 15 µm of the sample thickness) were acquired from the different focal planes for each specimen (one image in the proximity of the enamel, one in middle dentin, and one in deep dentin, close to the pulp). The fluorescence intensity emitted by the hydrolyzed fluorescein-conjugated gelatin was isolated and quantified using Image J software (ImageJ, U. S. National Institutes of Health, Bethesda, MD, USA). The relative amount of gelatinolytic activity was expressed as the integrated density of the pixels of the green fluorescence signal within the dentin structure. The evaluated data were homogenous and normally distributed and, therefore, were statistically analyzed using a one-way analysis of variance (ANOVA) and Bonferroni tests (Stata 12.0 software, StataCorp, College Station, TX, USA). The significance was preset at α = 0.05.

### 4.2. Gelatin Zymography

The zymographic analysis was performed according to the protocol by Mazzoni et al. [28]. Sound human extracted teeth (*n* = 8) were either stored in water at 4 °C and used within one week, or were washed under running water and were frozen immediately (−20 °C) and defrosted right before use. The teeth were ground free of enamel, pulpal soft tissue, and cementum. Dentin powder was obtained by trituration with a Retschmill (Reimiller, Reggio Emilia, Italy). Aliquots of 100 mg were prepared in duplicate for each group (Table 1) and the mineralized dentin powder was treated as follows: G1, application of 35% HP liquid per 10 min; G2, application of 35% HP liquid per 50 min; G3, application of 10% CP liquid per 252 min; G4, application of 10% CP liquid per 1260 min; and G5, no treatment (control group). The treatments proposed were performed with different exposure times corresponding to one week of each treatment (50 min of HP and 1260 min of CP) and the proportion between these treatments with 5 × lower exposure times (10 min of HP and 252 min of CP). The characteristics of 35% HP liquid and 10% CP liquid are described in Table 3.

For all groups, 1.8 mL of the extraction buffer (50 mM Tris-HCl, with a pH of 6.0, containing 5 mM CaCl_2_, 100 mM NaCl, 0.1% Triton X-100, 0.1% NONIDET P-40, 0.1 mM ZnCl_2_, and 0.02% NaN_3_ with an EDTA-free protease inhibitor cocktail) was added to the powder and was constantly agitated at 4 °C for 24 h, as well as being protected from the light. The samples were then sonicated in an ultrasound bath with water and ice for 10 min. The vials were centrifuged for 20 min at 4 °C (20,800× *g*) and the supernatants were collected. The protein content in the supernatants was concentrated using the Vivaspin centrifugal concentrator (10,000 KDa cut-off; Vivaspin Sartorius Stedim Biotech, Goettingen, Germany) for 20 min at 25 °C (15,000× *g* for 5 times). The total protein concentration of the dentin extracts was determined by the Bradford assay. Dentin protein aliquots (60 µg) were diluted with a Laemmli sample buffer in a 4:1 ratio. Electrophoresis was performed under non-reducing conditions using 10% sodium dodecyl sulphate-polyacrylamide gel (SDS-PAGE) containing 1 mg/mL fluorescent dye-labelled gelatin. Pre-stained low-range molecular weight SDS-PAGE standards (Bio-Rad, Hercules, CA, USA) were used as reference markers. After electrophoresis, the gels were washed for 1 h in 2% Triton X-100 and were incubated in a zymography activation buffer (50 mmol/L Tris-HCl and 5 mmol/L CaCl_2_, with a pH of 7.4) for 48 h. The proteolytic activity was evaluated and registered with a long-wave ultraviolet light scanner (ChemiDoc Universal Hood, Bio-Rad, Hercules, CA, USA). Gelatinase activities in the specimens were analyzed in duplicate. The densitometric evaluations of the bands obtained from the zymography were performed using the ImageJ software. 

## 5. Conclusions

The bleaching treatments performed on the enamel of sound teeth do not influence the dentinal enzymatic activity. On the other hand, when bleaching agents are applied on unprotected dentin tissue, the MMPs are more expressed, particularly when carbamide peroxide is used, and this effect is related to the exposure time.

## Figures and Tables

**Table 1:** Treatment groups (in-situ zymography).

		Method of Application	Number of Sessions
GROUP 1	Hydrogen peroxide 35%	Application of 50 min per session (the gel was renewed every 15 min)	4 *
GROUP 2	Carbamide peroxide 10%	Application of 180 min per session	21 *
GROUP 3	Control group	No treatment	-

* The number of bleaching sessions corresponds clinically to the entire treatment of each bleaching technique.

**Table 2:** Description of the bleaching products used.

Products	Description
35% hydrogen peroxide gel (Fórmula e Ação, São Paulo, Brazil)	35% hydrogen peroxide, thickener, vegetable extracts, amide, sequestering agent, glycol, and water. pH = 4.5.
10% carbamide peroxide gel (Fórmula e Ação)	10% carbamide peroxide, 3% potassium nitrate, 0.24% blood fluoride, humectant, thickener, preservative, mint aroma, and purified water qsp. pH = 6.8.

**Table 3:** Characteristics of 35% HP liquid and 10% CP liquid.

Products	Description
35% hydrogen peroxide liquid (Fórmula e Ação)	35% hydrogen peroxide, vegetable extracts, sequestering agent, glycol, and water. pH = 4.5.
10% carbamide peroxide liquid (Fórmula e Ação)	10% carbamide peroxide, 3% potassium nitrate, 0.24% sodium fluoride, humectant, preservative, and purified water qsp. pH = 6.8.

## Data Availability

The data presented in this study are available on request from the corresponding author.

## Sample Availability

Samples of the used bleaching compounds are available from the authors.
